# The Efficacy of Continuous Adductor Canal Block Oblique Approach to Postoperative Analgesia After Total Knee Arthroplasty: A Randomized, Double-Blinded, Controlled Clinical Trial

**DOI:** 10.1155/anrp/3267589

**Published:** 2025-09-30

**Authors:** Zhen Wan, Haiming Liao, Jinhuo Qin, Simin Tang, Jingjing Su, Jun Zhou

**Affiliations:** The Third Affiliated Hospital of Southern Medical University, 183 Zhongshan Avenue West, Tianhe District, Guangzhou, Guangdong, China

**Keywords:** adductor canal, anesthesiology, continuous catheter infusion, oblique approach, postoperative analgesia, total knee arthroplasty

## Abstract

**Background:**

Continuous adductor canal block (CACB) is a highly effective analgesic technique in total knee arthroplasty. The brachy axis approach (BA) is currently the common method used for CACB. Although CACB for BA offers advantages such as easy localization and operation due to clear anatomy, it has limitations such as short catheter placement and a high dislocation rate. These limitations can result in insufficient postoperative analgesia and impact patient rehabilitation. The perineural catheters placed parallel for CACB increases the difficulty of operation and the risk of puncture injury. In this study, the authors first applied the oblique approach (OA) to CACB and systematically compared the postoperative analgesic effects and adverse reactions of these two CACB methods with different puncture angles.

**Methods:**

Ninety subjects who underwent TKA were randomly assigned to receive ultrasound-guided CACB using either the OA or BA. The main outcome measured was the exercise maximum visual analog scale (VAS) score at 72 h after surgery. The main safety indicator was the rate of catheter dislocation. The data were recorded and analyzed using analysis of variance, with adjustments made for baseline characteristics.

**Results:**

There was no statistical difference in catheter placement time or postoperative resting VAS between the two groups. However, the postoperative exercise VAS and the incidence of postoperative adverse effects were lower in the OA group compared to the BA group.

**Conclusions:**

The OA of CACB applied after TKA improved the analgesic effect during the postoperative motor state compared to the BA and reduced the incidence of adverse reactions, without increasing the catheterization procedure time.

**Trial Registration:**

Chinese Clinical Trial Registry: ChiCTR2200059889

## 1. Introduction

With the aging population, there is an increasing incidence of knee osteoarthritis, and a growing number of patients require total knee arthroplasty (TKA) to restore knee function [1]. Post-TKA, the occurrence of moderate to severe pain is as high as 52% [2], resulting in limited knee mobility, while early postoperative rehabilitation exercises are crucial for knee function recovery [3, 4].

Common clinical methods for postoperative analgesia include intravenous analgesia, epidural analgesia, and nerve block analgesia using patient-controlled analgesia (PCA) [5–7]. However, these methods have limitations such as individual differences in efficacy and a high incidence of adverse reactions like nausea and vomiting with patient-controlled intravenous analgesia (PCIA) after TKA [8, 9]. Anticoagulant drugs are often used after TKA, and patient-controlled epidural analgesia (PCEA) is being phased out due to the increased risk of epidural hematoma [10, 11]. Patient-controlled nerve block analgesia (PCNA) can avoid adverse drug reactions associated with continuous postoperative use of intravenous analgesics and does not carry the risk of epidural hematoma. Consequently, PCNA has been widely adopted as a postoperative analgesia mode for TKA [12, 13]. The mainstream forms of PCNA for TKA include femoral nerve block (FNB) and adductor canal block (ACB) [14–16]. FNB primarily affects knee sensory and motor function, including the quadriceps muscles [4, 17], which can lead to quadriceps weakness and compromised balance during early functional exercises, increasing the risk of falls [18, 19]. ACB predominantly provides sensory blockade, targeting the saphenous nerve and other branches of the subsartorial plexus [20–22]. In contrast to FNB, ACB preserves quadriceps function, significantly reducing the risk of postoperative falls [23–25].

Continuous adductor canal block (CACB) offers longer-lasting analgesic effects compared to single ACB [26, 27]. However, the effectiveness of CACB guided by ultrasound may vary based on the catheter angle [28, 29]. The conventional approach for CACB is the brachy axis approach (BA) [30], which allows simultaneous visualization of blood vessels, nerves, and fascia, enabling precise localization and accurate catheterization positioning. Nevertheless, catheter dislocation remains a common issue [31]. The narrow anatomy of the adductor canal coupled with the relatively short catheter insertion in the BA approach can lead to reduced analgesic effects and affect patient postoperative rehabilitation. Studies have indicated that the dislocation rate of CACB using the BA approach can be as high as 37% [32]. Parallel placement for CACB catheter provided a lower postoperative catheter migration rate than the BA approach, but it has not been widely used in clinic because of the difficulty of localization. In this research, the authors propose the oblique approach (OA), which involves horizontally locating the adductor canal plane under ultrasound guidance, placing the ultrasound probe's M point on the lateral side, and rotating it 45° to align the catheter in the plane. The aim of this trial was to compare the effects of CACB using the OA and BA approaches on postoperative analgesia, catheter placement time, and the incidence of postoperative adverse reactions or complications.

In this single-center, randomized, parallel-group, double-blinded trial, the authors investigated the effects of the OA and BA approaches for CACB in patients undergoing primary unilateral TKA. The hypothesis was that CACB using the OA approach would provide better catheter position reliability and improved analgesia compared to the BA approach.

## 2. Methods

**Figure 1:**
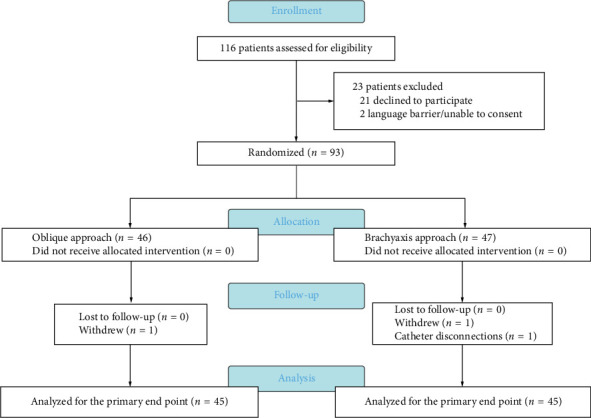
Legends: CONSORT study flow diagram.

Ethics Committee approval (The Third Affiliated Hospital of Southern Medical University, Guangdong Institute of Orthopedics, Guangzhou, Guangdong, China) was received on October 13, 2021. The procedures were conducted in accordance with the Helsinki Declaration-2013. This study was reported in accordance with the CONSORTreporting guidelines [33]. Ninety-three consecutive subjects scheduled for unilateral TKA were screened for eligibility after May 13, 2022. Ninety subjects provided written consent to participate in this single-center, randomized, parallel-group, double-blind trial. Inclusion criteria were as follows: age 18 years or older, first unilateral total knee replacement, American Society of Anesthesiology (ASA) grades I to III, and BMI of 18–35. Exclusion criteria encompassed contraindications related to peripheral nerve or nerve axis blocks, contraindications or allergies to test drugs or other anesthetics, coagulation disorders, confirmed/suspected abuse or self-use of narcotic sedatives and analgesia, existing neuropathy, previous lumbar spine surgery, and participation in other studies within 30 days. Additional exclusion criteria included unexpected intraoperative surgery or anesthesia accidents, catheter prolapse due to abnormal causes (e.g., violent pulling), conditions considered for exclusion by the investigator, subject rejection, and loss to follow-up. Random allocation was computer-generated by an independent statistician, with the sequence concealed in opaque envelopes that were opened by a research nurse (not involved in the trial) 1 h prior to surgery. The subjects were divided into two groups: the OA group (*n* = 45) and the BA group (*n* = 45). All CACB catheter placements were carried out by skilled regional anesthesia fellows under the direct supervision of senior investigators immediately after surgery. Apart from the investigators performing the blocks, all other researchers, anesthesia personnel, surgeons, physician assistants, nurses, and study participants were blinded to the randomization of each subject.  Figure 1 depicts the study flow diagram.

## 3. Standardized Anesthesia and Surgery Protocol

Upon entering the operating room, all subjects underwent routine vital signs monitoring, and multimode intravenous analgesia was initiated by establishing intravenous access (40 mg parecoxib sodium injection), as per our institution's standard practice and Ethics Committee requirements. Experienced anesthesiologists performed combined epidural-subarachnoid anesthesia using a lumbar anesthetic dose of 15-mg weight-specific ropivacaine (i.e., 1.5 mL of 1% ropivacaine diluted to 3 mL with cerebrospinal fluid). Continuous dexmedetomidine infusion was administered to all subjects during surgery for intraoperative sedation. Anesthesiologists maintained a constant rate of dexmedetomidine infusion to achieve a Ramsay sedation score of 5. Surgeons had the autonomy to choose the knee prosthesis, and prior to suturing, they performed local infiltration around the joint cavity using 20 mL of 0.25% bupivacaine and 10 mg of morphine. Adequate fluid rehydration was provided by the anesthesiologist during surgery, considering the duration of the procedure and physiological requirements to maintain subjects' vital sign stability.

## 4. Catheter Insertion and Management Protocol

**Figure 2:**
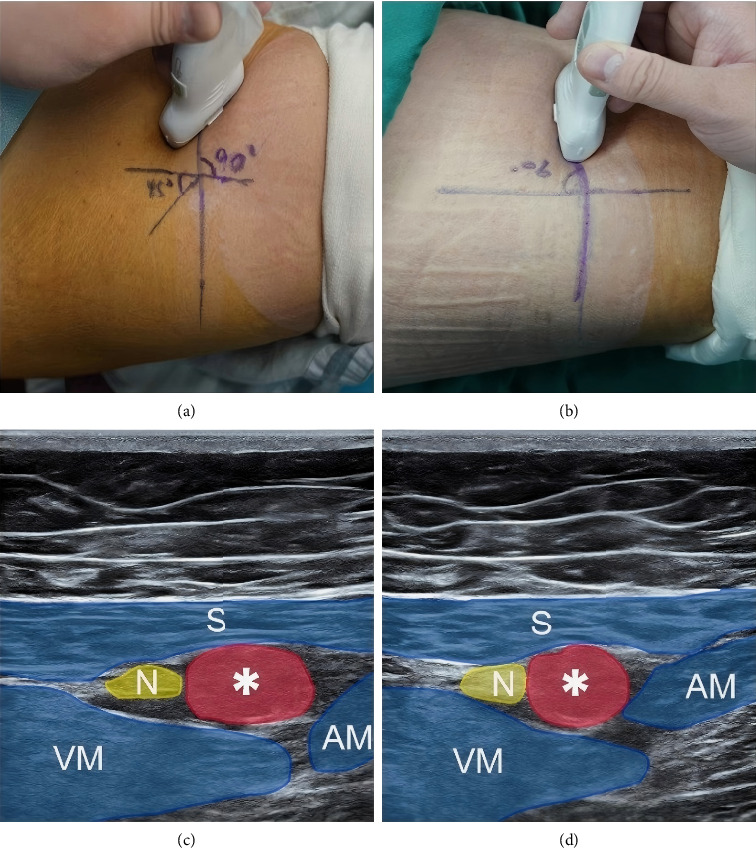
Legends: the oblique approach was inserted at the position of (a), and the brachyaxis approach inserted at the position of (b). Ultrasound images of the (c) oblique approach of adductor canal and (d) the brachyaxis approach of adductor canal. ^∗^, femoral artery; N, saphenous nerve; S, sartorius; VM, vastus Medialis; AM, adductor magnus.

All subjects received the same continuous nerve plexus catheter kit (Catheter Set and Cannulas for Continuous Plexus Anaesthesia 1.3 ∗ 80 MM, B. Braun Melsungen AG) and fixation method. The patient was placed in a supine position during catheter placement, and initial ultrasound scanning was performed using a 5–10 MHz linear ultrasound probe. The probe was positioned vertically in the medial mid-thigh to identify the vasto-adductor membrane (VAM). Above the VAM lies the sartorius muscle, which borders the vastus medialis laterally and the adductor magnus or adductor longus medially. In the BA group, after disinfection, the intraplate technique was employed to insert the needle from the lateral side into the fascial space between the sartorius muscle and the vastus medialis muscle. When the needle tip reached the sub-sartorius muscle and the superficial surface of the adductor membrane, 2–3 mL of local anesthetic solution (0.2% ropivacaine) was injected, creating a fluid space and opening the adductor canal. The needle was then passed through the adductor membrane into the adductor canal, and the initial dose of local anesthetic solution (20 mL of 0.2% ropivacaine) was injected to establish the adductor canal liquid space. After removing the needle core, a No. 20 peritoneal catheter was passed through the cannula tip 1–2 cm and then through the cannula itself. Ultrasonic positioning was used to guide the injection of 1 mL of 0.2% ropivacaine, ensuring proper catheter placement and fixation. In the OA group, after disinfection, the M point of the ultrasound probe was positioned on the outer side, and the ultrasonic probe was rotated 45° to visualize part of the adductor canal area. The intraplate technique was used to insert the needle from the lateral side into the fascial space between the sartorius muscle and the vastus medialis muscle. When the needle tip reached the sub-sartorius muscle and the superficial surface of the adductor membrane, 2–3 mL of local anesthetic solution (0.2% ropivacaine) was injected to create a liquid space and open the adductor canal. The needle was then passed through the adductor membrane into the adductor canal, and the initial dose of local anesthetic solution (20 mL of 0.2% ropivacaine) was injected to establish the adductor canal liquid space. After removing the needle core, a No. 20 peritoneal catheter was passed through the cannula tip 1–2 cm and then through the cannula itself. Ultrasonic positioning was used to guide the injection of 1 mL of 0.2% ropivacaine, ensuring proper catheter placement and fixation. Examples of both catheter locations are illustrated in Figure 2. The catheter was connected to a portable electronic infusion pump, and a 300-mL reservoir of 0.2% ropivacaine was configured. The infusion pump was set to a rate of 4 mL/h, with a bolus dose of 6 mL, PCA dose of 10 mL, locking time of 1 h, and a maximum dose of 20 mL/h. Infusion began immediately after catheter placement and remained connected until the trial ended on the third day.

## 5. Criteria Followed for Remedial Analgesia

If the patient experienced persistent pain in the innervation area of the Hunter's canal with a visual analog scale (VAS) score ≥ 4, and if the PCA did not reduce the VAS score to ≤ 3 while ultrasound confirmed catheter position changes, supplemental analgesia was administered. In such cases, intravenous administration of an equivalent morphine dose (a single dose equivalent to 2 mg morphine) was used for opioid analgesia supplementation. If the patient experienced pain in areas outside the Hunter's canal distribution, oral multimodal analgesia or analgesic injections on the ward were allowed.

## 6. Data Collection

During the follow-up period, the puncture sites were consistently covered with opaque white sterile gauze to preserve blinding throughout the study. Postoperative assessments were independently conducted by two research assistants blinded to group allocation. We recorded the time taken for catheter placement, including the following time points: ultrasound-guided localization and determination of the catheterization path (T1: positioning time), the time from needle insertion to cannula exit from the skin (T2: catheter insertion time), and the time required to determine the final catheter location using ultrasound (T3: location determination time). Patients reported their pain intensity using a VAS score ranging from 0 to 10, where 0 represents no pain and 10 represents the worst imaginable pain. Pain levels were recorded at rest and during active knee flexion or during the time up and go test (TUG) at 24, 48, and 72 h after surgery. The TUG measures the time taken to rise from a chair, walk a distance of 3 m, turn around, walk back, and sit down again. All patients used a high walker with arm support for the test. If the postoperative VAS score of a subject remained ≥ 4 and the pain originated from the Hunter's canal area, and if the pain did not improve after PCA supplementation (VAS score not < 3), the catheter position was examined using ultrasound. Localization was performed by injecting 0.9% normal saline through the catheter, and the fluid was checked to ensure it reached the adductor canal smoothly. If the injection failed to reach the adductor canal, it was considered as catheter dislocation (main safety indicators). Additional doses of ropivacaine through PCA and morphine equivalents were recorded for pain relief through the catheters up to 72 h postoperatively. Doses of oral opioid analgesics were standardized based on reported pain scores. Intravenous and oral opioid consumption during the 48 h after surgery were documented from the electronic medical record and converted to IV morphine equivalents. Subjects were asked to provide a dichotomous verbal assessment (“Satisfied” or “Unsatisfied”) of the quality of analgesia during the first and second postoperative days. Adverse reactions such as dizziness, severe vomiting, inadequate analgesia, excessive analgesia, respiratory depression, muscle weakness, lower limb vascular embolism, and other adverse events were recorded. Any potential adverse reactions (e.g., local anesthetic toxicity), complications (hematoma, infection, etc.), and any neurological deficits or other related problems during follow-up were also documented. Each day, the researchers directly inquired with the subjects about the occurrence of adverse reactions. Additionally, caregivers and researchers monitored any events that led to subject falls. All participants were consistently assigned to the same research team for follow-up evaluations throughout the study period. Standardized validated instruments (VAS/TUG) were employed, with dual independent data entry and cross-verification performed to minimize interobserver variability and associated measurement bias.

## 7. Sample Size Calculation

The sample size was determined based on an unpublished retrospective review conducted at our institution. With statistical differences in the exercise maximum VAS scores 72 h after surgery in the preliminary experiment, we chose the rate of catheter dislocation (the main safety indicator) to perform the sample size calculation. The minimum expected difference between the two groups was set at a postoperative tube displacement rate of 10%. With a type I error of 0.05 (*p* < 0.05) and a type II error of 0.2 (study efficacy of 80%), a sample size of 38 patients per group was required. To account for potential attrition, we aimed to recruit 45 patients per group.

## 8. Statistical Methodology

Statistical analyses were performed using SPSS for Windows version 26.0. A normality test was conducted to assess the data distribution, using the Kolmogorov–Smirnov test. Demographic and perioperative data were analyzed using the *t*-test and Pearson's chi-squared test. Pairwise comparisons were analyzed using the Wilcoxon test, with a significance level set at *p* < 0.05. The frequency of rescue analgesia, the incidence of complications related to regional block, and postoperative side effects were compared. Two-tailed analyses were conducted to determine statistical significance, with *p* < 0.05 considered statistically significant.

## 9. Data Quality

To ensure blinding of staff and research data collectors, wide opaque adhesive dressings were applied longitudinally over the anteromedian thigh, covering both potential insertion sites for the OA or BA catheters. The data collectors were not involved in intraoperative clinical care.

## 10. Results

A total of 116 patients were initially screened as eligible candidates, with 23 patients subsequently excluded. Ultimately, 93 subjects were randomly enrolled according to the study protocol, as depicted in Figure 1. There were no statistically significant differences observed in subject characteristics or surgical factors between the OA and BA groups (Table 1).

Among the 90 recruited subjects, 45 were assigned to the OA group and 45 to the BA group. In terms of safety indicators, there were no statistically significant differences in catheter displacement rates at 24, 48, or 72 h. However, differences were noted in the occurrence of related adverse reactions, primarily manifested as swelling and pain at the site of postoperative catheterization (*p* < 0.01) (Table 2).

Resting maximum VAS scores at postoperative 24 h (*p*=0.20) and 48 h (*p*=0.70) did not exhibit statistical differences. However, a statistical difference was observed at postoperative 72 h in resting maximum VAS scores between the BA (1 [0–2]) and OA subjects (1 [0–1]), *p* < 0.05. Additionally, in terms of exercise maximum VAS scores, statistical differences were noted at postoperative 24 h (BA: 2 [1,2] [1, 2] vs. OA: 1 [1,2] [1, 2]), postoperative 48 h (BA: 2 [1,2] [1, 2] vs. OA: 1 [1,2] [1, 2]), and postoperative 72 h (BA: 2 [1–3] [1–3] vs. OA: 1 [1,2] [1, 2]), all with *p* < 0.05 (Table 3).

**Figure 3:**
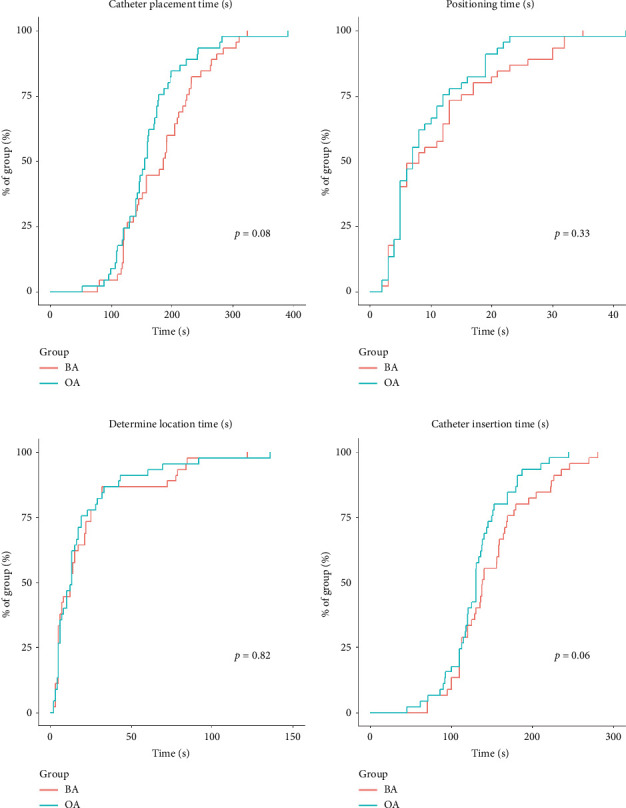
Legends: total completion time of the pipe placement operation and the completion time of each part of the operation. Represent the difficulty of the tube placement. Kaplan–Meier estimates of the cumulative percentages of subjects finishing the operation at each time point and subsequent time points.

**Figure 4:**
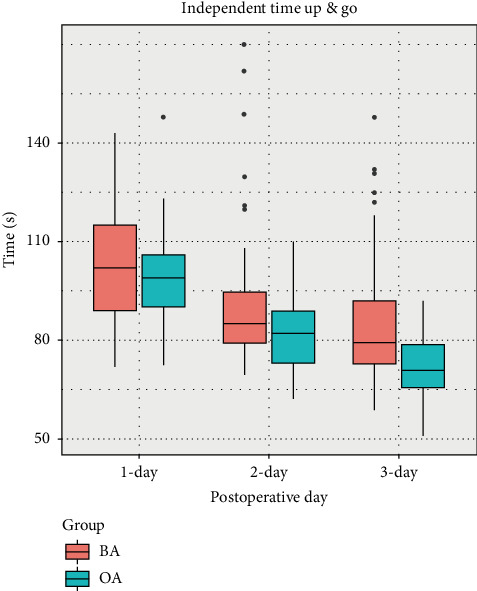
Legends: on the timed up and go test (independently stand, walk 3 m, return, and sit down) after total knee arthroplasty, using a four-legged walker. The time to perform the specified criteria as median (horizontal bar) with 25th to 75th (box) and 10th to 90th (whiskers) percentiles. Subjects with the oblique approach group get a much shorter TUG time. On the third postoperative day, the OA group in a median of 71 s (interquartile range, 66–78 s) compared with 79 s (72–91 s) for those with the BA group (*p* < 0.01).

Regarding secondary outcome measures, there were no statistically significant differences in T1 time (T1) between the BA (8 [5–15] [5–15]s) and OA subjects (7 [5–12] [5–12]s) (median [interquartile range]); *p*=0.52. Similarly, no significant differences were found in T2 time (T2): BA (138 [113–170]s) vs OA (130 [113–150] s), *p*=0.16, or in T3 time (T3): BA (13s) vs OA (13s), *p*=0.89 (Figure 3). No differences were observed in the TUG at 24 h after surgery, while statistically significant differences emerged at 48 h (*p*=0.04) and 72 h (*p* < 0.01) after surgery(Figure 4). Although there were no differences in the consumption of salvage analgesic drugs, variations in PCA drug usage between the two groups were observed (*p* < 0.01). Patient satisfaction at 72 h also exhibited statistical differences (Table 3).

## 11. Discussion

In this randomized, double-blind, single-center study, a total of 90 patients undergoing TKA were included. There were no statistically significant differences observed in the time required for catheter placement at the three steps (T1, T2, and T3). This may be attributed to the simultaneous visualization of blood vessels, muscles, and aponeuroses during CACB by the OA, which does not pose additional challenges for experienced anesthesiologists. Both approaches provided satisfactory analgesia at rest after CACB, likely due to the successful targeting of the adductor canal using different methods in both groups. The OA approach resulted in better analgesia during active movement, potentially because the catheter tip inserted via OA was directed distally, allowing for greater spread of local anesthetics to the knee joint. The oblique positioning of the catheter may facilitate greater diffusion of the local anesthetic not only to the saphenous nerve but also to the branch of the vastus medialis nerve, thereby enhancing analgesic efficacy during early mobilization [34]. Concurrently, this may enable better pain control during postoperative rehabilitation activities by reaching the popliteal joint and anesthetizing the posterior branch of the obturator nerve and popliteal plexus.

In our trial, the catheter displacement rate was 0% in the OA group and 11% in the block adductor (BA) group. Although the difference is not statistically significant, catheter dislocation occurred only in the BA group and not in the OA group, suggesting potentially greater stability of catheter positioning with the OA. The longer catheter length achievable with OA may reduce the likelihood of catheter dislodgment from the Hunter canal during use and facilitate locating the adductor canal catheter tip.

CACB by OA resulted in a lower incidence of adverse effects (24.4% in the OA group vs. 51% in the BA group). Adverse reactions in the OA group primarily involved numbness in the innervated area, which is an acceptable side effect of nerve blocks. In the BA group, five cases experienced drug fluid leakage at the puncture site, two cases had slight blood infiltration, and ten cases reported medial thigh pain, none of which occurred in the OA group. The narrower internal space of the Hunter's canal in the BA approach may contribute to a higher likelihood of extravasation and muscle pain near the catheter location compared to OA. This discrepancy may explain the longer TUG time and increased PCA drug dose observed after 48 h in the BA group. Additionally, the BA approach involves traversing more muscle tissue, increasing the risk of bleeding, hematoma development, and infection [35–37]. Fortunately, no serious adverse effects such as falls, infections, or local anesthetic poisoning occurred in the 90 subjects. The study also allowed surgeons to perform infiltration around the knee capsule, enhancing the analgesic effect on posterior knee pain [38–40]. Quadriceps muscle strength and the timing of the patient's first ambulation were not evaluated in this study due to the advantages of CACB not affecting postoperative quadriceps strength and variations in postoperative rehabilitation practices.

This trial demonstrated that CACB for postoperative analgesia can be performed without compromising efficacy, prolonging operation time, or increasing difficulty. The OA approach, which involves rotating the ultrasound probe 45° when placing the M point on the lateral side, offers a more reliable catheter placement location with a lower risk of adverse reactions compared to the BA approach, which places the ultrasound probe vertically on the thigh.

As a single-center randomized controlled trial, the results of this study should be further confirmed through multicenter trials and expanded sample sizes to account for intergroup variability. Additionally, the findings are specific to the local anesthetic types, concentrations, additional volumes, and basal rates used in this investigation. The primary endpoint in this study was the VAS score at 72 h post-surgery, aligning with the earliest normal discharge time at our center. Long-term postoperative rehabilitation outcomes were not observed, but future studies plan to include extended observation periods (up to 3 months after surgery) to assess physical and psychological parameters and gain a better understanding of the effects of CACB via OA on long-term TKA rehabilitation.

## 12. Conclusion

This comparative study evaluated the differential effects of two CACB approaches—OA versus conventional BA—utilizing distinct puncture angles for post-TKA analgesia. Results demonstrated that although no statistically significant differences existed in catheterization procedure time or resting VAS scores between cohorts, the OA group exhibited significantly reduced movement-evoked VAS scores and lower incidence of adverse reactions. Consequently, we conclude that OA of CACB applied after TKA optimizes perioperative analgesia during ambulation while reducing complication rates, without prolonging catheterization procedure time relative to the BA technique.

## Figures and Tables

**Table 1:** Anthropomorphic and prerandomization surgical characteristics of the study subjects.

Characteristic	Randomization group			*p*value
	All subject	Brachyaxis approach	Oblique approach	
	(*n* = 90)	(*n* = 45)	(*n* = 45)	
Age (yr)	67 ± 7	67 ± 7	67 ± 7	0.99
Sex (M/F) (%)	17 (19%)/73 (81%)	39 (87%)/6 (13%)	34 (76%)/11 (24%)	0.18
Height (cm)	158 (153–162)	156 (153–160)	158 (155–165)	0.12
Weight (kg)	65 (59–70)	66 (60–70)	65 (57–70)	0.47
BMI (kg/m^2^)	26 (23–28)	26 (25–28)	25 (23–28)	0.24
ASA (%)	2 (2–2)	2 (2–2)	2 (2–2)	0.60
Operated knee (L/R) (%)	44 (49%)/46 (51%)	22 (49%)/23 (51%)	24 (53%)/21 (47%)	0.67
Duration of surgery (min)	95 (85–119)	95 (85–115)	95 (85–120)	0.83
Worst pain during resting state (VAS)	2 (1-2)	2 (1–3)	2 (1-2)	0.27
Worst pain during exercise state (VAS)	6 (6-7)	7 (6-7)	6 (6-7)	0.07

*Note:* Results expressed as median (inter-quartile range); A *p* value of < 0.05 was considered statistically significant.

Abbreviation: BMI, body mass index.

**Table 2:** Main and secondary safety indexes.

	All subject (*n* = 90)	Brachyaxis approach (*n* = 45)	Oblique approach (*n* = 45)	*p* value
Main safety indicators				
Inadvertent catheter dislodgement, day 1 (%)	0 (0)	0 (0)	0 (0)	—
Inadvertent catheter dislodgement, day 2 (%)	3 (3.3)	3 (6.67)	0 (0)	0.24
Inadvertent catheter dislodgement, day 3 (%)	5 (5.6)	5 (11.11)	0 (0)	0.07
Secondary safety indicators				
Untoward effect (%)	34 (37.8)	23 (51.11)	11 (24.44)	< 0.01^∗^
Local skin numbness or hypesthesia (%)	27 (30.0)	16 (35.56)	11 (24.44)	0.25
Fluid leakage at catheter site (%)	8 (8.9)	7 (15.56)	1 (2.22)	0.06
Muscle aches (%)	10 (11.1)	10 (22.22)	0 (0)	< 0.01^∗^
Fall (%)	0 (0)	0 (0)	0 (0)	—
Poisoning by local anesthetic (%)	0 (0)	0 (0)	0 (0)	—
Catheter insertion infection (%)	0 (0)	0 (0)	0 (0)	—

*Note:* Data are reported as *n* (%); days refer to postoperative day.

^∗^A *p* value of < 0.05 was considered statistically significant.

**Table 3:** Primary and secondary endpoints.

	All subject (*n* = 90)	Brachyaxis approach (*n* = 45)	Oblique approach (*n* = 45)	*p* value
Primary endpoints				
Catheter placement time (s)	159 (127, 209)	185 (126–225)	154 (129–177)	0.08
Positioning time (s)	7 (5–13)	8 (5–15)	7 (5–12)	0.52
Catheter insertion time (s)	134 (113–166)	138 (113–170)	130 (113–150)	0.16
Determine location time (s)	13 (5–23)	13 (5–25)	13 (5–19)	0.89
Postoperative pain; maximum VAS				
Resting state, 24 h	1 (0-1)	1 (0-1)	0 (0-1)	0.20
Resting state, 48 h	1 (0-1)	1 (0-1)	1 (0-1)	0.70
Resting state, 72 h	1 (0-1)	1 (0–2)	1 (0-1)	0.02
Exercise state, 24 h	1 (1-2)	2 (1-2)	1 (1-2)	< 0.01^∗^
Exercise state, 48 h	2 (1-2)	2 (1-2)	1 (1-2)	0.04^∗^
Exercise state, 72 h	1 (1-2)	2 (1–3)	1 (1-2)	< 0.01^∗^
Secondary endpoints				
Timed up and go, day 1 (TUG1) (s)	99 (89–111)	102 (89–115)	99 (90–106)	0.28
Timed up and go, day 2 (TUG2) (s)	84 (77–92)	85 (79–95)	82 (73–89)	0.04^∗^
Timed up and go, day 3 (TUG3) (s)	75 (69–83)	79 (72–91)	71 (66–78)	< 0.01^∗^
Total ropivacaine administered of PCA (mg)	1.8 (0.3–3.3)	2.4 (1.2–4.8)	1.2 (0–2.4)	< 0.01^∗^
Postoperative morphine equivalents (mg)	0 (0–0)	0 (0–0)	0 (0–0)	0.05
Patient satisfaction, day 1 (%)	90 (100)	45 (100.00)	45 (100.00)	—
Patient satisfaction, day 2 (%)	86 (95.6)	41 (91.11)	45 (100.00)	0.13
Patient satisfaction, day 3 (%)	84 (93.3)	39 (86.67)	45 (100.00)	0.04^∗^

*Note:* Results expressed as median (inter-quartile range); Days refer to postoperative day.

Abbreviation: VAS, visual analog scale.

^∗^A *p* value of < 0.05 was considered statistically significant.

## Data Availability

Data available upon request from the authors. The data that support the findings of this study are available from the corresponding author upon reasonable request.

## Nomenclature

CACB: Continuous adductor canal block
TKA: Total knee arthroplasty
BA: Brachy axis approach
OA: oblique approach
PCA: Patient-controlled analgesia
PCIA: Patient-controlled intravenous analgesia
PCEA: Patient-controlled epidural analgesia
PCNA: Patient-controlled nerve block analgesia
FNB: Femoral nerve block
ACB: Adductor canal block
ChiCTR: Chinese Clinical Trial Registry
ASA: American Society of Anesthesiology
VAM: Vasto-adductor membrane
VAS: Visual analog score
TUG: Time up and go test
